# Identification of conserved gene expression features between murine mammary carcinoma models and human breast tumors

**DOI:** 10.1186/gb-2007-8-5-r76

**Published:** 2007-05-10

**Authors:** Jason I Herschkowitz, Karl Simin, Victor J Weigman, Igor Mikaelian, Jerry Usary, Zhiyuan Hu, Karen E Rasmussen, Laundette P Jones, Shahin Assefnia, Subhashini Chandrasekharan, Michael G Backlund, Yuzhi Yin, Andrey I Khramtsov, Roy Bastein, John Quackenbush, Robert I Glazer, Powel H Brown, Jeffrey E Green, Levy Kopelovich, Priscilla A Furth, Juan P Palazzo, Olufunmilayo I Olopade, Philip S Bernard, Gary A Churchill, Terry Van Dyke, Charles M Perou

**Affiliations:** 1 Lineberger Comprehensive Cancer Center; 2 Curriculum in Genetics and Molecular Biology, University of North Carolina at Chapel Hill, Chapel Hill, NC 27599, USA; 3 Department of Cancer Biology, University of Massachusetts Medical School, Worcester, MA 01605, USA; 4 Department of Biology and Program in Bioinformatics and Computational Biology, University of North Carolina at Chapel Hill, Chapel Hill, NC 27599, USA; 5 The Jackson Laboratory, Bar Harbor, ME 04609, USA; 6 Department of Genetics, University of North Carolina at Chapel Hill, Chapel Hill, NC 27599, USA; 7 Department of Oncology, Lombardi Comprehensive Cancer Center, Georgetown University, Washington, DC 20057, USA; 8 Department of Pathology, University of Chicago, Chicago, IL 60637, USA; 9 Department of Pathology, University of Utah School of Medicine, Salt Lake City, UT 84132, USA; 10 Baylor College of Medicine, Houston, TX 77030, USA; 11 Transgenic Oncogenesis Group, Laboratory of Cancer Biology and Genetics; 12 Chemoprevention Agent Development Research Group, National Cancer Institute, Bethesda, MD 20892, USA; 13 Department of Pathology, Thomas Jefferson University, Philadelphia, PA 19107, USA; 14 Section of Hematology/Oncology, Department of Medicine, Committees on Genetics and Cancer Biology, University of Chicago, Chicago, IL 60637, USA; 15 Department of Pathology and Laboratory Medicine, University of North Carolina at Chapel Hill, Chapel Hill, NC 27599, USA

## Abstract

**Background:**

Although numerous mouse models of breast carcinomas have been developed, we do not know the extent to which any faithfully represent clinically significant human phenotypes. To address this need, we characterized mammary tumor gene expression profiles from 13 different murine models using DNA microarrays and compared the resulting data to those from human breast tumors.

**Results:**

Unsupervised hierarchical clustering analysis showed that six models (TgWAP-*Myc*, TgMMTV-*Neu*, TgMMTV-*PyMT*, TgWAP-*Int3*, TgWAP-*Tag*, and TgC3(1)-*Tag*) yielded tumors with distinctive and homogeneous expression patterns within each strain. However, in each of four other models (TgWAP-*T*_*121*_, TgMMTV-*Wnt1*, *Brca1*^*Co*/*Co*^;TgMMTV-*Cre*;*p53*^+/- ^and DMBA-induced), tumors with a variety of histologies and expression profiles developed. In many models, similarities to human breast tumors were recognized, including proliferation and human breast tumor subtype signatures. Significantly, tumors of several models displayed characteristics of human basal-like breast tumors, including two models with induced *Brca1 *deficiencies. Tumors of other murine models shared features and trended towards significance of gene enrichment with human luminal tumors; however, these murine tumors lacked expression of estrogen receptor (ER) and ER-regulated genes. TgMMTV-*Neu *tumors did not have a significant gene overlap with the human HER2+/ER- subtype and were more similar to human luminal tumors.

**Conclusion:**

Many of the defining characteristics of human subtypes were conserved among the mouse models. Although no single mouse model recapitulated all the expression features of a given human subtype, these shared expression features provide a common framework for an improved integration of murine mammary tumor models with human breast tumors.

## Abstract 2

Comparison of mammary tumor gene-expression profiles from thirteen murine models using microarrays and with that of human breast tumors showed that many of the defining characteristics of human subtypes were conserved among mouse models.

## Background

Global gene expression analyses of human breast cancers have identified at least three major tumor subtypes and a normal breast tissue group [1]. Two subtypes are estrogen receptor (ER)-negative with poor patient outcomes [2,3]; one of these two subtypes is defined by the high expression of HER2/ERBB2/NEU (HER2+/ER-) and the other shows characteristics of basal/myoepithelial cells (basal-like). The third major subtype is ER-positive and Keratin 8/18-positive, and designated the 'luminal' subtype. This subtype has been subdivided into good outcome 'luminal A' tumors and poor outcome 'luminal B' tumors [2,3]. These studies emphasize that human breast cancers are multiple distinct diseases, with each of the major subtypes likely harboring different genetic alterations and responding distinctly to therapy [4,5]. Further similar investigations may well identify additional subtypes useful in diagnosis and treatment; however, such research would be accelerated if the relevant disease properties could be accurately modeled in experimental animals. Signatures associated with specific genetic lesions and biologies can be causally assigned in such models, potentially allowing for refinement of human data.

Significant progress in the ability to genetically engineer mice has led to the generation of models that recapitulate many properties of human cancers [6]. Mouse mammary tumor models have been designed to emulate genetic alterations found in human breast cancers, including inactivation of TP53, BRCA1, and RB, and overexpression of MYC and HER2/ERBB2/NEU. Such models have been generated through several strategies, including transgenic overexpression of oncogenes, expression of dominant interfering proteins, targeted disruption of tumor suppressor genes, and by treatment with chemical carcinogens [7]. While there are many advantages to using the mouse as a surrogate, there are also potential caveats, including differences in mammary physiologies and the possibility of unknown species-specific pathway differences. Furthermore, it is not always clear which features of a human cancer are most relevant for disease comparisons (for example, genetic aberrations, histological features, tumor biology). Genomic profiling provides a tool for comparative cancer analysis and offers a powerful means of cross-species comparison. Recent studies applying microarray technology to human lung, liver, or prostate carcinomas and their respective murine counterparts have reported commonalities [8-10]. In general, each of these studies focused on a single or few mouse models. Here, we used gene expression analysis to classify a large set of mouse mammary tumor models and human breast tumors. The results provide biological insights among and across the mouse models, and comparisons with human data identify biologically and clinically significant shared features.

## Results

### Murine tumor analysis

To characterize the diversity of biological phenotypes present within murine mammary carcinoma models, we performed microarray-based gene expression analyses on tumors from 13 different murine models (Table 1) using Agilent microarrays and a common reference design [1]. We performed 122 microarrays consisting of 108 unique mammary tumors and 10 normal mammary gland samples (Additional data file 1). Using an unsupervised hierarchical cluster analysis of the data (Additional data file 2), murine tumor profiles indicated the presence of gene sets characteristic of endothelial cells, fibroblasts, adipocytes, lymphocytes, and two distinct epithelial cell types (basal/myoepithelial and luminal). Grouping of the murine tumors in this unsupervised cluster showed that some models developed tumors with consistent, model-specific patterns of expression, while other models showed greater diversity and did not necessarily group together. Specifically, the TgWAP-*Myc*, TgMMTV-*Neu*, TgMMTV-*PyMT*, TgWAP-*Int3 *(*Notch4*), TgWAP-*Tag *and TgC3(1)-*Tag *tumors had high within-model correlations. In contrast, tumors from the TgWAP-*T*_*121*_, TgMMTV-*Wnt1*, *Brca1*^*Co*/*Co*^;TgMMTV-Cre;*p53*^+/-^, and DMBA-induced models showed diverse expression patterns. The *p53*^-/- ^transplant model tended to be homogenous, with 4/5 tumors grouping together, while the *Brca1*^+/-^;*p53*^+/- ^ionizing radiation (IR) and *p53*^+/- ^IR models showed somewhat heterogeneous features between tumors; yet, 6/7 *Brca1*^+/-^;*p53*^+/- ^IR and 5/7 *p53*^+/- ^IR were all present within a single dendrogram branch.

**Table 1:** Summary of mouse mammary tumor models

Tumor model	No. of tumors	Specificity of lesions	Experimental oncogenic lesion(s)	Strain	Reference
TgWAP-*Myc*	13	WAP*	cMyc overexpression	FVB	[60]
TgWAP-*Int3*	7	WAP	Notch4 overexpression	FVB	[61]
TgWAP-*T*_*121*_	5	WAP	pRb, p107, p130 inactivation	B6D2	[37]
TgWAP-*T*_*121*_	2	WAP	pRb, p107, p130 inactivation	BALB/cJ	[37]
TgWAP-*Tag*	5	WAP	SV40 L-T (pRb, p107, p130, p53, p300 inactivation, others); SV40 s-t	C57Bl/6	[62]
TgC3(1)-*Tag*	8	C3(1)^†^	SV40 L-T (pRb, p107, p130, p53, p300 inactivation, others); SV40 s-t	FVB	[63]
TgMMTV-*Neu*	10	MMTV^‡^	Unactivated rat Her2 overexpression	FVB	[64]
TgMMTV-*Wnt1*	11	MMTV	Wnt 1 overexpression	FVB	[65]
TgMMTV-*PyMT*	7	MMTV	Py-MT (activation of Src, PI-3' kinase, and Shc)	FVB	[66]
TgMMTV-*Cre*;*Brca1*^*Co*/*Co*^;*p*53^+/-^	10	MMTV	Brca1 truncation mutant; p53 heterozygous null	C57Bl/6	[67]
*p*53^-/-^transplanted	5	None	p53 inactivation	BALB/cJ	[68]
Medroxyprogesterone-DMBA-induced	11	None	Random DMBA-induced	FVB	[69]
*p*53^+/-^irradiated	7	None	p53 heterozygous null, random IR induced	BALB/cJ	[70]
*Brca1*^+/-^;*p*53^+/-^irradiated	7	None	Brca1 and p53 heterozygous null, random IR induced	BALB/cJ	[1]

*WAP, *whey acidic protein *promoter, commonly restricted to lactating mammary gland luminal cells. ^†^C3(1), 5' flanking region of the C3(1) component of the rat prostate steroid binding protein, expressed in mammary ductal cells. ^‡^MMTV, mouse mammary tumor virus promoter, often expressed in virgin mammary gland epithelium, induced with lactation; often expressed at ectopic sites (for example, lymphoid cells, salivary gland, others).

**Figure 1:**
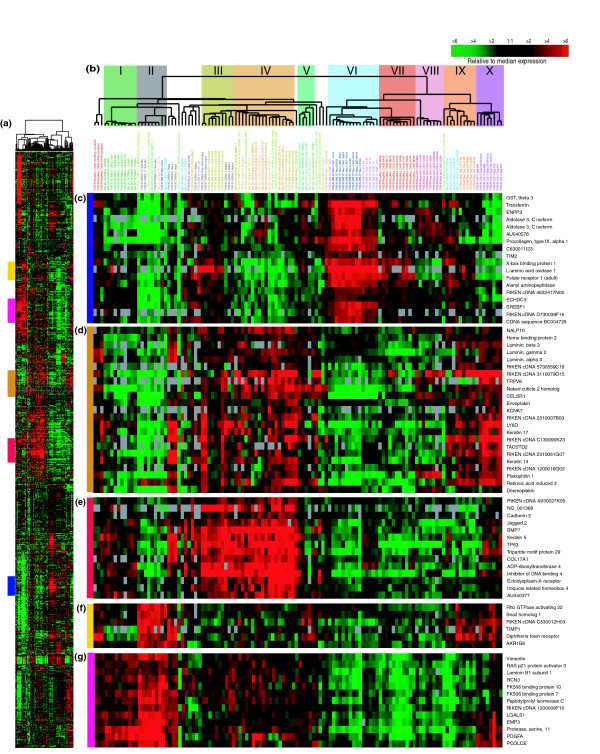
Mouse models intrinsic gene set cluster analysis. **(a) **Overview of the complete 866 gene cluster diagram. **(b) **Experimental sample associated dendrogram colored to indicate ten groups. **(c) **Luminal epithelial gene expression pattern that is highly expressed in TgMMTV-*PyMT*, TgMMTV-*Neu*, and TgWAP-*myc *tumors. **(d) **Genes encoding components of the basal lamina. **(e) **A second basal epithelial cluster of genes, including *Keratin *5. **(f) **Genes expressed in fibroblast cells and implicated in epithelial to mesenchymal transition, including *snail homolog *1. **(g) **A second mesenchymal cluster that is expressed in normals. See Additional data file 2 for the complete cluster diagram with all gene names.

As with previous human tumor studies [1,3], we performed an 'intrinsic' analysis to select genes consistently representative of groups/classes of murine samples. In the human studies, expression variation for each gene was determined using biological replicates from the same patient, and the 'intrinsic genes' identified by the algorithm had relatively low variation within biological replicates and high variation across individuals. In contrast, in this mouse study we applied the algorithm to groups of murine samples defined by an empirically determined correlation threshold of > 0.65 using the dendrogram from Additional data file 2. This 'intrinsic' analysis yielded 866 genes that we then used in a hierarchical cluster analysis (Figure 1 and Additional data file 3 for the complete cluster diagram). This analysis identified ten potential groups containing five or more samples each, including a normal mammary gland group (Group I) and nine tumor groups (designated Groups II-X).

**Figure 2:**
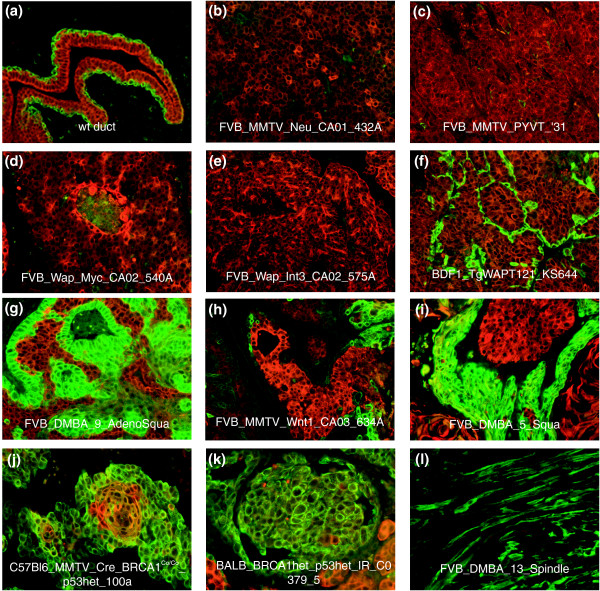
Immunofluorescence staining of mouse samples for basal/myoepithelial and luminal cytokeratins. **(a) **Wild-type (wt) mammary gland stained for Keratins 8/18 (red) and Keratin 5 (green) shows K8/18 expression in luminal epithelial cells and K5 expression in basal/myoepithelial cells. **(b-f) **Mouse models that show luminal-like gene expression patterns stained with K8/18 (red) and K5 (green). **(g-k) **Tumor samples that show basal-like, or mixed luminal and basal characteristics by gene expression, stained for K8/18 (red) and K5 (green). **(j) **A subset of *Brca1*^*Co*/*Co*^;TgMMTV-Cre;*p*53^+/-^tumors showing nodules of K5/K8/18 double positive cells. **(l) **A splindloid tumor stained for K8/18 (red) and smooth muscle actin (green).

In general, these ten groups were contained within four main categories that included (Figure 1b, left to right): the normal mammary gland samples (Group I) and tumors with mesenchymal characteristics (Group II); tumors with basal/myoepithelial features (Groups III-V); tumors with luminal characteristics (Groups VI-VIII); and tumors containing mixed characteristics (Groups IX and X). Group I contained all normal mammary gland samples, which showed a high level of similarity regardless of strain, and was characterized by the high expression of basal/myoepithelial (Figure 1e) and mesenchymal features, including *vimentin *(Figure 1g). Group II samples were derived from several models (2/10 *Brca1*^*Co*/*Co*^;TgMMTV-Cre;*p53*^+/-^, 3/11 DMBA-induced, 1/5 *p53*^-/- ^transplant, 1/7 *p53*^+/- ^IR, 1/10 TgMMTV-*Neu *and 1/7 TgWAP-*T*_*121*_) and also showed high expression of mesenchymal features (Figure 1g) that were shared with the normal samples in addition to a second highly expressed mesenchymal-like cluster that contained *snail homolog 1 *(a gene implicated in epithelial-mesenchymal transition [11]), the latter of which was not expressed in the normal samples (Figure 1f). Two TgWAP-*Myc *tumors at the extreme left of the dendrogram, which showed a distinct spindloid histology, also expressed these mesenchymal-like gene features. Further evidence for a mesenchymal phenotype for Group II tumors came from Keratin 8/18 (K8/18) and smooth muscle actin (SMA) immunofluorescence (IF) analyses, which showed that most spindloid tumors were K8/18-negative and SMA-positive (Figure 2l).

The second large category contained Groups III-V, with Group III (4/11 DMBA-induced and 5/11 *Wnt1*), Group IV (7/7 Brca1^+/-^;*p53*^+/- ^IR, 4/10 *Brca1*^*Co*/*Co*^;TgMMTV-Cre;*p53*^+/-^, 4/6 *p53*^+/- ^IR and 3/11 *Wnt1*) and Group V (4/5 *p53*^-/- ^transplant and 1/6 *p53*^+/- ^IR), showing characteristics of basal/myoepithelial cells (Figure 1d, e). These features were encompassed within two expression patterns. One cluster included *Keratin 14*, *17 *and *LY6D *(Figure 1d); *Keratin 17 *is a known human basal-like tumor marker [1,12], while *LY6D *is a member of the Ly6 family of glycosylphosphatidylinositol (GPI)-anchored proteins that is highly expressed in head and neck squamous cell carcinomas [13]. This cluster also contained components of the basement membrane (for example, *Laminins*) and hemidesmosomes (for example, *Envoplakin *and *Desmoplakin*), which link the basement membrane to cytoplasmic keratin filaments. A second basal/myoepithelial cluster highly expressed in Group III and IV tumors and a subset of DMBA tumors with squamous morphology was characterized by high expression of *ID4*, *TRIM29*, and *Keratin 5 *(Figure 1e), the latter of which is another human basal-like tumor marker [1,12]. This gene set is expressed in a smaller subset of models compared to the set described above (Figure 1d), and is lower or absent in most Group V tumors. As predicted by gene expression data, most of these tumors stained positive for *Keratin 5 *(K5) by IF (Figure 2g-k).

The third category of tumors (Groups VI-VIII) contained many of the 'homogenous' models, all of which showed a potential 'luminal' cell phenotype: Group VI contained the majority of the TgMMTV-*Neu *(9/10) and TgMMTV-*PyMT *(6/7) tumors, while Groups VII and VIII contained most of the TgWAP-*Myc *tumors (11/13) and TgWAP-*Int3 *samples (6/7), respectively. A distinguishing feature of these tumors (in particular Group VI) was the high expression of *XBP1 *(Figure 1c), which is a human luminal tumor-defining gene [14-17]. These tumors also expressed tight junction structural component genes, including *Occludin*, *Tight Junction Protein 2 *and *3*, and the luminal cell K8/18 (Additional data file 2). IF for K8/18 and K5 confirmed that these tumors all exclusively expressed K8/18 (Figure 2b-f).

Finally, Group IX (1/10 *Brca1*^*Co*/*Co*^;TgMMTV-Cre;*p53*^+/-^, 4/7 TgWAP-*T*_*121 *_tumors and 5/5 TgWAP-*Tag *tumors) and Group X (8/8 TgC3(1)-*Tag*) tumors were present at the far right and showed 'mixed' characteristics; in particular, the Group IX tumors showed some expression of luminal (Figure 1c), basal (Figure 1d) and mesenchymal genes (Figure 1f), while Group X tumors expressed basal (Figure 1e,f) and mesenchymal genes (Figure 1f,g).

IF analyses showed that, as in humans [12,18], the murine basal-like models tended to express K5 while the murine luminal models expressed only K8/18. However, some of the murine basal-like models developed tumors that harbored nests of cells of both basal (K5+) and luminal (K8/18+) cell lineages. For example, in some TgMMTV-*Wnt1 *[19], DMBA-induced (Figure 2g,i), and *Brca1*-deficient strain tumors, distinct regions of single positive K5 and K8/18 cells were observed within the same tumor. Intriguingly, in some *Brca1*^*Co*/*Co*^;TgMMTV-Cre;*p53*^+/- ^samples, nodules of double-positive K5 and K8/18 cells were identified, suggestive of a potential transition state or precursor/stem cell population (Figure 2j), while in some TgMMTV-*Wnt1 *(Figure 2h) [19] and *Brca1*-deficient tumors, large regions of epithelioid cells were present that had little to no detectable K5 or K8/18 staining (data not shown).

**Figure 3:**
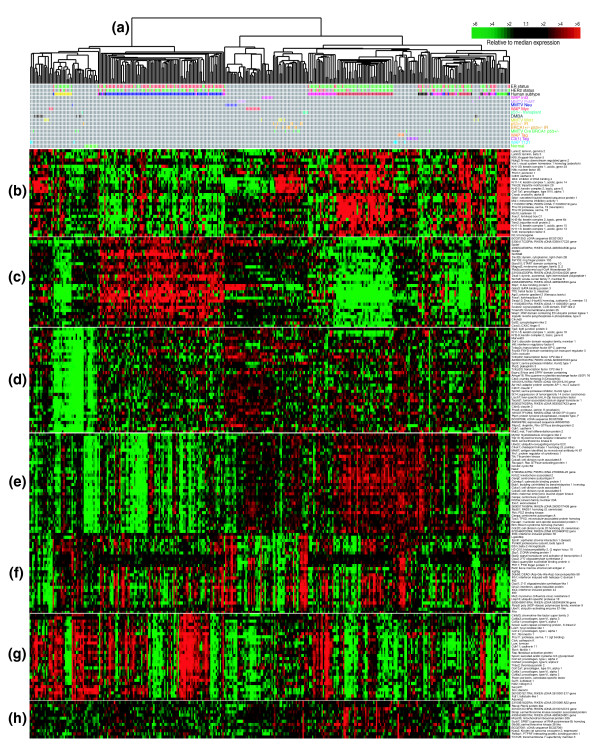
Unsupervised cluster analysis of the combined gene expression data for 232 human breast tumor samples and 122 mouse mammary tumor samples. **(a) **A color-coded matrix below the dendrogram identifies each sample; the first two rows show clinical ER and HER2 status, respectively, with red = positive, green = negative, and gray = not tested; the third row includes all human samples colored by intrinsic subtype as determined from Additional data file 6; red = basal-like, blue = luminal, pink = HER2+/ER-, yellow = claudin-low and green = normal breast-like. The remaining rows correspond to murine models indicated at the right. **(b) **A gene cluster containing basal epithelial genes. **(c) **A luminal epithelial gene cluster that includes *XBP1 *and *GATA3*. **(d) **A second luminal cluster containing *Keratins *8 and 18. **(e) **Proliferation gene cluster. **(f) **Interferon-regulated genes. **(g) **Fibroblast/mesenchymal enriched gene cluster. **(h) **The *Kras2 *amplicon cluster. See Additional data file 5 for the complete cluster diagram.

The reproducibility of these groups was evaluated using 'consensus clustering' (CC) [20]. CC using the intrinsic gene list showed strong concordance with the results sown in Figure 1 and supports the existence of most of the groups identified using hierarchical clustering analysis (Additional data file 4). However, our further division of some of the CC-defined groups appears justified based upon biological knowledge. For instance, hierarchical clustering separated the normal mammary gland samples (Group I) and the histologically distinct spindloid tumors (Group II), which were combined into a single group by CC. Groups VI (TgMMTV-*Neu *and *PyMT*) and VII (TgWAP-*Myc*) were likewise separated by hierarchical clustering, but CC placed them into a single category. CC was also performed using all genes that were expressed and varied in expression (taken from Additional data file 2), which showed far less concordance with the intrinsic list-based classifications, and which often separated tumors from individual models into different groups (Figure 3c, bottom most panel); for example, the TgMMTV-*Neu *tumors were separated into two or three different groups, whereas these were distinct and single groups when analyzed using the intrinsic list. This is likely due to the presence or absence of gene expression patterns coming from other cell types (that is, lymphocytes, fibroblasts, and so on) in the 'all genes' list, which causes tumors to be grouped based upon qualities not coming from the tumor cells [1].

### Mouse-human combined unsupervised analysis

The murine gene clusters were reminiscent of gene clusters identified previously in human breast tumor samples. To more directly evaluate these potential shared characteristics, we performed an integrated analysis of the mouse data presented here with an expanded version of our previously reported human breast tumor data. The human data were derived from 232 microarrays representing 184 primary breast tumors and 9 normal breast samples also assayed on Agilent microarrays and using a common reference strategy (combined human datasets of [21-23] plus 58 new patients/arrays). To combine the human and mouse datasets, we first used the Mouse Genome Informatics database to identify well-annotated mouse and human orthologous genes. We then performed a distance weighted discrimination correction, which is a supervised analysis method that identifies systematic differences present between two datasets and makes a global correction to compensate for these global biases [24]. Finally, we created an unsupervised hierarchical cluster of the mouse and human combined data (Figure 3 and Additional data file 5 for the complete cluster diagram).

This analysis identified many shared features, including clusters that resemble the cell-lineage clusters described above. Specifically, human basal-like tumors and murine *Brca1*^+/-^;*p53*^+/-^;IR, *Brca1*^*Co*/*Co*^;TgMMTV-Cre;*p53*^+/-^, TgMMTV-*Wnt1*, and some DMBA-induced tumors were characterized by the high expression of *Laminin gamma 2*, *Keratins 5*, *6B*, *13*, *14*, *15*, *TRIM29*, *c-KIT *and *CRYAB *(Figure 3b), the last of which is a human basal-like tumor marker possibly involved in resistance to chemotherapy [25]. As described above, the *Brca1*^+/-^;*p53*^+/-^;IR, some *Brca1*^*Co*/*Co*^;TgMMTV-Cre;*p53*^+/^, DMBA-induced, and TgMMTV-*Wnt1 *tumors stained positive for K5 by IF, and human basal-like tumors tend to stain positive using a K5/6 antibody [1,12,18,26], thus showing that basal-like tumors from both species share K5 protein expression as a distinguishing feature.

The murine and human 'luminal tumor' shared profile was not as similar as the shared basal profile, but did include the high expression of *SPDEF*, *XBP1 *and *GATA3 *(Figure 3c), and both species' luminal tumors also stained positive for K8/18 (Figure 2 and see [18]). For many genes in this luminal cluster, however, the relative level of expression differed between the two species. For example, some genes were consistently high across both species' tumors (for example, *XBP1*, *SPDEF *and *GATA3*), while others, including *TFF*, *SLC39A6*, and *FOXA1*, were high in human luminal tumors and showed lower expression in murine tumors. Of note is that the human luminal epithelial gene cluster always contains the *Estrogen*-*Receptor *(*ER*) and many estrogen-regulated genes, including *TFF1 *and *SLC39A6 *[22]; since most murine mammary tumors, including those profiled here, are ER-negative, the apparent lack of involvement of ER and most ER-regulated genes could explain the difference in expression for some of the human luminal epithelial genes that show discordant expression in mice.

Several other prominent and noteworthy features were also identified across species, including a 'proliferation' signature that includes the well documented proliferation marker Ki-67 (Figure 3e) [1,27,28] and an interferon-regulated pattern (Figure 3f) [27]. The proliferation signature was highest in human basal-like tumors and in the murine models with impaired pRb function (that is, Group IX and X tumors). Currently, the growth regulatory impact of interferon-signaling in human breast tumors is not understood, and murine models that share this expression feature (TgMMTV-*Neu*, TgWAP-Tag, *p53*^-/- ^transplants, and spindloid tumors) may provide a model for future studies of this pathway. A fibroblast profile (Figure 3g) that was highly expressed in murine samples with spindloid morphology and in the TgWAP-*Myc *'spindloid' tumors was also observed in many human luminal and basal-like tumors; however, on average, this profile was expressed at lower levels in the murine tumors, which is consistent with the relative epithelial to stromal cell proportions seen histologically.

Through these analyses we also discovered a potential new human subtype (Figure 3, top line-yellow group, and Additional data file 6). This subtype, which was apparent in both the human only and mouse-human combined dataset, is referred to as the 'claudin-low' subtype and is characterized by the low expression of genes involved in tight junctions and cell-cell adhesion, including *Claudins 3*, *4*, *7*, *Occludin*, and *E-cadherin *(Figure 3d). These human tumors (*n *= 13) also showed low expression of luminal genes, inconsistent basal gene expression, and high expression of lymphocyte and endothelial cell markers. All but one tumor in this group was clinically ER-negative, and all were diagnosed as grade II or III infiltrating ductal carcinomas (Additional data file 7 for representative hematoxylin and eosin images); thus, these tumors do not appear to be lobular carcinomas as might be predicted by their low expression of *E-cadherin*. The uniqueness of this group was supported by shared mesenchymal expression features with the murine spindloid tumors (Figure 3g), which cluster near these human tumors and also lack expression of the *Claudin *gene cluster (Figure 3d). Further analyses will be required to determine the cellular origins of these human tumors.

### A common region of amplification across species

The murine C3(1)-*Tag *tumors and a subset of human basal-like tumors showed high expression of a cluster of genes, including *Kras2*, *Ipo8*, *Ppfibp1*, *Surb*, and *Cmas*, that are all located in a syntenic region corresponding to human chromosome 12p12 and mouse chromosome 6 (Figure 3h). *Kras2 *amplification is associated with tumor progression in the C3(1)-*Tag *model [29], and haplo-insufficiency of *Kras2 *delays tumor progression [30]. High co-expression of *Kras2*-linked genes prompted us to test whether DNA copy number changes might also account for the high expression of *Kras2 *among a subset of the human tumors. Indeed, 9 of 16 human basal-like tumors tested by quantitative PCR had increased genomic DNA copy numbers at the *KRAS2 *locus; however, no mutations were detected in *KRAS2 *in any of these 16 basal-like tumors. In addition, van Beers *et al*. [31] reported that this region of human chromosome 12 is amplified in 47% of *BRCA1*-associated tumors by comparative genomic hybridization analysis; *BRCA1*-associated tumors are known to exhibit a basal-like molecular profile [3,32]. In cultured human mammary epithelial cells, which show basal/myoepithelial characteristics [1,33], both high oncogenic H-ras and SV40 Large T-antigen expression are necessary for transformation [34]. Taken together, these findings suggest that amplification of *KRAS2 *may either influence the cellular phenotype or define a susceptible target cell type for basal-like tumors.

### Mouse-human shared intrinsic features

**Figure 4:**
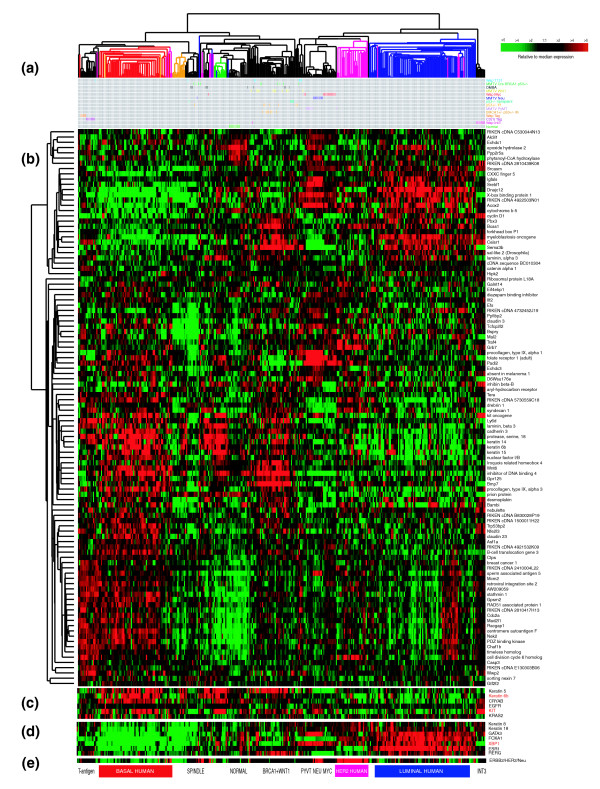
Cluster analysis of mouse and human tumors using the subset of genes common to both species intrinsic lists (106 total genes). **(a) **Experimental sample associated dendrogram color coded according to human tumor subtype and with a matrix below showing murine tumor origins. **(b) **The complete 106 gene cluster diagram. **(c) **Close-up of genes known to be important for human basal-like tumors. **(d) **Close-up of genes known to be important for human luminal tumors, including ER. **(e) **Expression pattern of HER2/ERBB2/NEU.

To simultaneously classify mouse and human tumors, we identified the gene set that was in common between a human breast tumor intrinsic list (1,300 genes described in Hu *et al*. [21]) and the mouse intrinsic list developed here (866 genes). The overlap of these two lists totaled 106 genes, which when used in a hierarchical clustering analysis (Figure 4) identifies four main groups: the leftmost group contains all the human basal-like, 'claudin-low', and 5/44 HER2+/ER- tumors, and the murine C3(1)-*Tag*, TgWAP-*Tag*, and spindloid tumors. The second group (left to right) contains the normal samples from both humans and mice, a small subset (6/44) of human HER2+/ER- and 10/92 luminal tumors, and a significant portion of the remaining murine basal-like models. By clinical criteria, nearly all human tumors in these two groups were clinically classified as ER-negative.

The third group contains 33/44 human HER2+/ER- tumors and the murine TgMMTV-*Neu*, MMTV-PyMT and TgWAP-*Myc *samples. Although the human HER2+/ER- tumors are predominantly ER-negative, this comparative genomic analysis and their keratin expression profiles as assessed by immunohistochemistry, suggests that the HER2+/ER- human tumors are 'luminal' in origin as opposed to showing basal-like features [18]. The fourth and right-most group is composed of ER-positive human luminal tumors and, lastly, the mouse TgWAP-*Int3 *(*Notch4*) tumors were in a group by themselves. These data show that although many mouse and human tumors were located on a large dendrogram branch that contained most murine luminal models and human HER2+/ER- tumors, none of the murine models we tested showed a strong human 'luminal' phenotype that is characterized by the high expression of *ER*, *GATA3*, *XBP1 *and *FOXA1*. These analyses suggest that the murine luminal models like MMTV-*Neu *showed their own unique profile that was a relatively weak human luminal phenotype that is missing the ER-signature. Presented at the bottom of Figure 4 are biologically important genes discussed here, genes previously shown to be human basal-like tumor markers (Figure 4c), human luminal tumor markers, including ER (Figure 4d), and HER2/ERBB2/NEU (Figure 4e).

### A comparison of gene sets defining human tumors and murine models

We used a second analysis method called gene set enrichment analysis (GSEA) [35] to search for shared relationships between human tumor subtypes and murine models. For this analysis, we first performed a two-class unpaired significance analysis of microarray (SAM) [36] analysis for each of the ten murine groups defined in Figure 1, and obtained a list of highly expressed genes that defined each group. Next, we performed similar analyses using each human subtype versus all other human tumors. Lastly, the murine lists were compared to each human subtype list using GSEA, which utilizes both gene list overlap and gene rank (Table 2). We found that the murine Groups IX (*p *= 0.004) and X (*p *= 0.001), which comprised tumors from pRb-deficient/p53-deficient models, shared significant overlap with the human basal-like subtype and tended to be anti-correlated with human luminal tumors (*p *= 0.083 and 0.006, respectively). Group III murine tumors (TgMMTV-*Wnt1 *mostly) significantly overlapped human normal breast samples (*p *= 0.008), possibly due to the expression of both luminal and basal/myoepithelial gene clusters in both groups. Group IV (*Brca1-deficient *and *Wnt1*) showed a significant association (*p *= 0.058) with the human basal-like profile. The murine Group VI (TgMMTV-*Neu *and TgMMTV-*PyMT*) showed a near significant association (*p *= 0.078) with the human luminal profile and were anti-correlated with the human basal-like subtype (*p *= 0.04). Finally, the murine Group II spindloid tumors showed significant overlap with human 'claudin-low' tumors (*p *= 0.001), which further suggests that this may be a distinct and novel human tumor subtype.

**Table 2:** Gene set enrichment analysis of the ten murine groups versus five human subtypes

		Basal-like		Luminal		HER2+/ER-		Normal		Claudin-low	
						
Mouse class	No. of genes	*p *value	*p *value	*p *value	*p *value	*p *value	*p *value	*p *value	*p *value	*p *value	*p *value
**Is class**											
I	1,882	-	-	0.4625	0.8755	0.5388	0.9137	0.1659	0.5628	0.0048	0.1028
II	912	-	-	-	-	0.5867	0.9609	-	-	0.0021	**0.001**
III	143	0.5289	0.9048	-	-	0.5285	0.9047	0	**0.008**	-	-
IV	1,019	0	**0.0581**	-	-	-	-	-	-	-	-
V	34	-	-	0.8492	0.998	0.9324	0.999	-	-	0.0427	0.09274
VI	820	-	-	0.0062	0.0783	0.3536	0.7864	0.8653	0.9769	-	-
VII	851	0.1258	0.3768	-	-	0.5616	0.9137	-	-	-	-
VIII	236	0.1449	0.6098	0.3483	0.8205	-	-	0.01878	0.2349	-	-
IX	462	0.0019	**0.004**	-	-	0.56	0.9509	-	-	-	-
X	338	0	**0.001**	-	-	0.9275	0.998	-	-	-	-
**Is not class**											
I	1,882	0.0128	0.1662	-	-	-	-	-	-	-	-
II	912	0.3996	0.8348	0.8601	0.999	-	-	0.3602	0.7655	-	-
III	143	-	-	0.3178	0.7259	-	-	-	-	0.7628	0.991
IV	1,019	-	-	0.1833	0.6516	0.398	0.8427	0.2241	0.7255	0.1453	0.6116
V	34	0.86	1	-	-	-	-	0.0656	0.1653	-	-
VI	820	0	**0.04**	-	-	-	-	-	-	0.1043	0.4444
VII	851	-	-	0.1733	0.5151	-	-	0.5403	0.9128	0.1628	0.5215
VIII	236	-	-	-	-	0.1131	0.5305	-	-	0.6427	0.961
IX	462	-	-	0.04305	0.0833	-	-	0.022	**0.037**	0.2612	0.5936
X	338	-	-	0.02236	0.0682	-	-	0.1313	0.3717	0.5437	0.9489

Statistically significant findings are highlighted in bold. NOM = nominal.

We also performed a two-class unpaired SAM analysis using each mouse model as a representative of a pathway perturbation using the transgenic 'event' as a means of defining groups. Models that yielded a significant gene list (false discovery rate (FDR) = 1%) were compared to each human subtype as described above (Additional data file 8). The models based upon SV40 T-antigen (all C3(1)-Tag and WAP-Tag tumors) shared significant overlap with the human basal-like tumors (*p *= 0.002) and were marginally anti-correlated with the human luminal class. The BRCA1 deficient models (all Brca1^+/-^;*p53*^+/- ^IR and *Brca1*^*Co*/*Co*^;TgMMTV-Cre;*p53*^+/- ^tumors) were marginally significant with human basal-like tumors (*p *= 0.088). The TgMMTV-*Neu *tumors were nominally significant (before correction for multiple comparisons) with human luminal tumors (*p *= 0.006) and anti-correlated with human basal-like tumors (*p *= 0.027).

The two most important human breast tumor biomarkers are ER and HER2; therefore, we also analyzed these data relative to these two markers. Of the 232 human tumors assayed here, 137 had ER and HER2 data assessed by immunohistochemistry and microarray data. As has been noted before [3,18,21], there is a very high correlation between tumor intrinsic subtype and ER and HER2 clinical status (*p *< 0.0001): for example, 81% of ER+ tumors were of the luminal phenotype, 63% of HER2+ tumors were classified as HER2+/ER-, and 80% of ER- and HER2- tumors were of the basal-like subtype. Using GSEA, we compared the ten mouse classes as defined in Figure 1 (Additional data file 9) and the mouse model-based gene lists (Additional data file 10) to the human data/gene lists that were obtained by performing supervised analyses based upon human ER and HER2 status (please note that analyses using HER2 status alone (that is, HER2+ versus HER2-), and ER+ and HER2+ versus others were not included as human classes because HER2 status alone yielded genes on only the HER2 amplicon, and the ER+ and HER2+ classification did not yield a significant gene list). We found that the murine Groups IX (*p *= 0.009) and X (*p *= 0.003) tumors shared significant overlap with ER- HER2- human tumors and were significantly anti-correlated with human ER+ tumors (*p *= 0.024 and 0.043, respectively). Group VI murine samples (TgMMTV-*Neu *and TgMMTV-*PyMT*) likewise showed the same trend of enrichment with ER+ human tumors and anti-correlation with the ER- HER2- class. Although not perfect, these GSEA results are consistent with our observations from Figures 1 and 3 and again demonstrate that the basal-like profile is robustly shared between humans and mice, while the luminal profile shows some shared and some distinct features across species.

## Discussion

Gene expression profiling of murine tumors and their comparison to human tumors identified characteristics relevant to individual murine models, to murine models in general, and to cancers of both species. First was the discovery that some murine models developed highly similar tumors within models, while others showed heterogeneity in expression and histological phenotypes. For the homogenous models, the study of progression or response to therapy is simplified because confounding variation across individuals is low. An example of this consistency even extended to secondary events that occurred within the TgC3(1)-*Tag *model, where many tumors shared the amplification and high expression of *Kras2 *(Figure 3h) - a feature also evident in a subset of human basal-like tumors.

In contrast to the 'homogenous' models are models such as TgWAP-*T*_*121*_, DMBA-induced and *Brca1*^*Co*/*Co*^;TgMMTV-Cre;*p53*^+/-^, where individual tumors within a given model often showed different gene expression profiles and histologies. It is likely that these models fall into one of three scenarios that could explain their heterogeneity: the first, represented by the TgWAP-*T*_*121 *_model [37], is that the transgene is responsible only for initiating tumorigenesis, leaving progression events to evolve stochastically and with longer latency periods. Such a model would likely give rise to different tumor subtypes depending on the subsequent pathways that are disrupted during tumor progression. A second possibility is that the initiating event generates genomic instability such that multiple distinct pathways can be affected by the experimental causal event, which may be the mechanism in the *Brca1*-inactivation tumors. The third scenario is that the target cell of transformation is a multi-potent progenitor with the ability to undergo differentiation into multiple epithelial lineages, or even mesenchymal lineages (for example, DMBA-induced and *Brca1*^*Co*/*Co*^;TgMMTV-Cre;*p53*^+/-^); support for this hypothesis comes from Keratin IF analyses in which, even within a histologically homogenous tumor, two types of epithelial cells are present (Figures 2g-k). The presence of subsets of individual cells positive for markers of two epithelial cell types also supports this possibility (Figure 2j). Alternative hypotheses include the possibility that multiple cell types sustain transforming events, and also that extensive non-cell-autonomous tissue responses occur. Regardless of the paradigm of transformation for these heterogeneous models, the study of progression or therapeutic response will best be accomplished by first sub-setting by subtype, and then focusing on biological phenotypes.

There are at least two major applications for genomic comparisons between human tumors and their potential murine counterparts. First, such studies should identify those models that contain individual and/or global characteristics of a particular class of human tumors. Examples of important global characteristics identified here include the classification of murine and human tumors into basal and luminal groups. It appears as if four murine models developed potential luminal-like tumors (TgMMTV-*Neu*, TgMMTV-*PyMT*, TgWAP-*Myc*, and TgWAP-*Int3*), which is not surprising since both MMTV and WAP are thought to direct expression in differentiated alveolar/luminal cells [38,39]; however, it should be noted that the luminal profile across species was not statistically significant, likely due to the lack of ER and ER-regulated genes in the murine luminal tumors. Several murine models did show expression features consistent with human basal-like tumors, including the TgC3(1)-*Tag*, TgWAP-*Tag *and *Brca1*-deficient models. The SV40 T-antigen used in the TgC3(1)-*Tag *and TgWAP-*Tag *models inactivates p53 and RB, which also appear to be two likely events that occur in human basal-like tumors because these tumors are known to harbor *p53 *mutations [2], have high mitotic grade and the highest expression of proliferation genes (Figure 3) [2,3], which are known E2F targets [40]. The proliferation signature in human breast cancers is itself prognostic [41], and is also predictive of response to chemotherapy [42]. These data suggest that human basal-like tumors might have impairment of RB function and highlight an important shared feature of murine and human mammary carcinomas.

The finding that *Brca1 *loss (coincident with *p53 *mutation) in mice gives rise to tumors with a basal-like phenotype is notable because humans carrying *BRCA1 *germline mutations also develop basal-like tumors [3,32], and most human *BRCA1 *mutant tumors are p53-deficient [43,44]. These data suggest a conserved predisposition of the basal-like cell type, or its progenitor cell, to transform as a result of *BRCA1*, *TP53*, and *RB*-pathway loss. Most DMBA-induced carcinomas also showed basal-like cell lineage features, suggesting that this cell type is also susceptible to DMBA-mediated tumorigenesis. Finally, some TgMMTV-*Wnt1 *tumors showed a combination of basal-like and luminal characteristics by gene expression, which is consistent with the observation that tumors of this model generally contain cells from both mammary epithelial lineages [45].

The second major purpose of comparative studies is to determine the extent to which analyses of murine models can inform the human disease and guide further discovery. An example of murine models informing the human disease is encompassed by the analysis of the new potential human subtype discovered here (that is, claudin-low subtype). Further analysis will be necessary to confirm whether this is a *bona fide *subtype; however, the statistically significant gene overlap with a histologically distinct subset of murine tumors suggests it is a distinct biological entity. A second example of the murine tumors guiding discovery in humans was the common association of a K-Ras containing amplicon in a subset of human basal-like tumors and in the murine basal-like TgC3(1)-*Tag *strain tumors.

An important caveat to all comparative studies is that there are clear biological differences between mice and humans, which may or may not directly impact disease mechanisms. A potential example of inherent species difference could be the aforementioned biology associated with ER and its downstream pathway. In humans, ER is highly expressed in luminal tumors [1], with the luminal phenotype being characterized by the high expression of some genes that are ER-regulated like *PR *and *RERG *[22], and other luminal genes that are likely GATA3-regulated, including *AGR2 *and *K8/18 *[46]. In mice, ER expression is low to absent in all the tumors we tested, as is the expression of most human ER-responsive genes. This finding is consistent with previous reports that most late-stage murine mammary tumors are ER-negative ([47] and references within). However, it should be noted that two human luminal tumor-defining genes (*XBP1 *and *GATA3 *[46], were both highly expressed in murine luminal tumors (Additional data file 2). Taken together, these data suggest that the human 'luminal' profile may actually be a combination of at least two profiles, one of which is ER-regulated and another of which is GATA3-regulated; support for a link between *GATA3 *and luminal cell origins comes from *GATA3 *loss studies in mice where the selective loss of *GATA3 *in the mammary gland resulted in either a lack of luminal cells, or a significant decrease in the number and/or maturation of luminal cells [48,49]. These results suggest that, in the mouse models tested here, the ER-regulated gene cassette that is present in human luminal tumors is missing, and that the GATA3-mediated luminal signature remains. Due to the partial luminal tumor signature in mice, we believe that the murine luminal models, including TgMMTV-*Neu *profiled here, best resemble human luminal tumors and more specifically possibly luminal B tumors, which are luminal tumors that express low amounts of ER and show a poor outcome [2,3,21]. While human HER2+/ER- subtype tumors and the murine TgMMTV-*Neu*, TgMMTV-*PyMT*, and TgWAP-*Myc *fall next to each other in the intrinsic-shared cluster (Figure 4), all of the other data argue against this association. A few murine ER-positive mammary tumor models have been developed [50-53]; however, none of these models were analyzed here.

Of note, many expression patterns detected in this study were observed in only one species (Additional data file 5), and it is possible that some of these differences may arise from technical limitations rather than reflect important biological differences. Comparison between two expression datasets, especially when derived from different species, remains a technical challenge. Thus, we acknowledge the possibility that artifacts may have been introduced depending on the data analysis methodology. However, we are confident that the analyses described here identified many common and biologically relevant clusters, including a proliferation, basal epithelial, interferon-regulated and fibroblast signature, thus showing that the act of data combining across species did retain important features present within the individual datasets. There are many murine models of breast cancer that we did not look at in this study and many more will be developed. Like the 13 models we discussed here, we would expect that some of these models will have overlapping gene expression patterns with human subtypes while others will not. We believe that additional studies with larger numbers of samples, including more diversity from each species, is warranted. These analyses do confirm the notion that there is not a single murine model that perfectly represents a human breast cancer subtype; however, the murine models do show shared features with specific human subtypes and it is these commonalties that will lay the groundwork for many future studies.

## Materials and methods

### Murine and human tumors

The murine tumor samples were obtained from multiple participating investigators, who all maintained the mice and harvested the murine tumors in the 0.5-1 cm stage following internationally recognized guidelines. The details concerning strain background, promoter, transgene, and specific alleles, and so on, are provided in Additional data file 1. All human tumor samples were collected from fresh frozen primary breast tumors using Institutional Review Board (IRB)-approved protocols and were profiled as described in [21-23]. The clinical and pathological information for these human samples can be obtained at the University of North Carolina Microarray Database (UMD) [54].

### Microarray experiments

Total RNA was collected from murine tumors, and wild-type mammary samples of both FVB and BALB/c inbred strains. RNA was purified using the RNeasy Mini Kit (Qiagen Inc., Valencia, CA, USA) according to the manufacturer's protocol using 20-30 mg tissue. RNA integrity was assessed using the RNA 6000 Nano LabChip kit followed by analysis using a Bioanalyzer (Agilent Technologies Inc., Santa Clara, CA, USA). Total RNA (2.5 μg) was reverse transcribed, amplified and labeled with Cy5 using a Low RNA Input Amplification kit (Agilent). The common reference RNA sample for these experiments consisted of total RNA harvested from equal numbers of C57Bl6/J and 129 male and female day 1 pups (a gift from Dr Cam Patterson, UNC). The reference RNA was reverse transcribed, amplified, and labeled with Cy3. The amplified sample and reference were co-hybridized overnight to Agilent Mouse Oligo Microarrays (G4121A). They were then washed and scanned on a GenePix 4000B scanner (Molecular Devices Corporation, Sunnyvale, CA, USA), analyzed using GenePix 4.1 software and uploaded into our database where a Lowess normalization is automatically performed.

### Microarray data analysis

All primary microarray data are available from the UMD [54], and at the Gene Expression Omnibus under the series GSE3165 (mouse and new human data), GSE1992, GSE2740 and GSE2741 (previously published human data) [55]. The genes for all analyses were filtered by requiring the Lowess normalized intensity values in both channels to be > 30. The log_2 _ratio of Cy5/Cy3 was then reported for each gene. In the final dataset, only genes that reported values in 70% or more of the samples were included. The genes were median centered and then hierarchical clustering was performed using Cluster v2.12 [56]. For the murine unsupervised analysis, and human-mouse unsupervised cluster analyses, we filtered for genes that varied at least three-fold or more, in at least three or more samples. Average linkage clustering was performed on genes and arrays and cluster viewing and display was performed using JavaTreeview v1.0.8 [57].

### Mouse Intrinsic gene set analysis

Intrinsic 'groups' of experimental samples were chosen based upon having a Pearson correlation value of 0.65 or greater from the unsupervised clustering analysis of the 122 murine samples. The analysis was performed using the Intrinsic Gene Identifier v1.0 by Max Diehn/Stanford University [1]. Technical replicates were removed from the file and the members of every highly correlated node were given identical class numbers, giving every sample that fell outside the 0.65 correlation cut-off a class of their own. Using these criteria, 16 groups of samples were identified (see Additional data file 1 for these groups) and a list of 866 'intrinsic' genes was selected using the criteria of one standard deviation below the mean intrinsic gene value. A human intrinsic list of 1,300 genes was created using a subset of 146 of the 232 samples used here, and is described in Hu *et al*. [21].

### Consensus clustering

CC [20] was performed locally using Gene Pattern 1.3.1 (built Jan 6, 2005), which was downloaded from the Broad Institute distribution website [58]. Analyses were performed on the mouse dataset with all genes, and just with intrinsic genes separately. Ranges for the number of K clusters (or the focused number of classes) were from 2 to 15 to evaluate a wide range of possible groups. Using a Euclidian distance measure with average linkage, we re-sampled 1,000 times with both column and row normalization.

### Combining murine and human expression datasets

Orthologous genes were reported by Mouse Genome Informatics (MGI 3.1) of The Jackson Laboratory. For both the human and murine datasets, Locus Link IDs assigned to Agilent oligo probe ID numbers were used to assign to MGI ID numbers. In cases where a single gene was represented by multiple probes, the median value of the redundant probes was used. This led to a total of orthologous pairings of 14,680 Agilent probes. Prior to combining the two datasets, each was column standardized to N(0,1), row median centered, and probe identifiers were converted to MGI IDs. The intersection of mouse and human MGI identifiers from genes that passed filters (same as used above) in both datasets yielded 7,907 orthologous genes in the total combined dataset. This dataset was next corrected for systemic biases using distance weighted discrimination [24]. Finally, the combined dataset was used for an average linkage hierarchical clustering analysis.

### Gene set enrichment analysis

We took the 232 human samples and classified them as basal-like, luminal, HER2+/ER-, claudin-low, and normal breast-like according to a clustering analysis of the human dataset only (Additional data file 6), using the new intrinsic/UNC human gene list developed in Hu *et al*. [21]. Second, the murine samples were also classified based upon their clustering pattern in Figure 1 that used the mouse intrinsic gene list, and were assigned to Groups I-X. Two-class unpaired SAM analysis was performed for each murine class separately versus all other classes using an FDR of 1% [36], resulting in 10 class-specific gene lists. Using only the set of highly expressed genes that were associated with each analysis (and ignoring the genes whose low expression correlated with a given class), GSEA [35] was performed in R (v. 2.0.1) using the GSEA R package [59]. The ten murine gene sets were then compared to each human subtype-ranked gene set and significant enrichments reported. For statistical strength of these enrichments, GSEA uses family wise error rate (FWER) to correct for multiple testing and FDR to reduce false positive reporting. The parameters used for all GSEA were: nperm = 1,000, weighted.score.type = 1, nom.p.val.threshold = -1, fwer.p.val.threshold = -1, fdr.q.val.threshold = 0.25, topgs = 12, adjust.FDR.q.val = FALSE, gs.size.threshold.min = 25, gs.size.threshold.max = 2,000, reverse.sign = FALSE, preproc.type = 0, random.seed = 3,338, perm.type = 0, fraction = 1, replace = FALSE.

### Immunofluorescence

Paraffin-embedded sections (5 μm thick) were processed using standard immunostaining methods. The antibodies and their dilution were α-cytokeratin 5 (K5, 1:8,000, PRB-160P, Covance, Berkeley, CA, USA), and α-cytokeratins 8/18 (Ker8/18, 1:450, GP11, Progen Biotecknik, Heidelberg, Germany). Briefly, slides were deparaffinized and hydrated through a series of xylenes and graded ethanol steps. Heat-mediated epitope retrieval was performed in boiling citrate buffer (pH 6.0) for 15 minutes, then samples cooled to room temperature for 30 minutes. Secondary antibodies for immunofluorescence were conjugated with Alexa Fluor-488 or -594 fluorophores (1:200, Molecular Probes, Invitrogen, Carlsbad, CA, USA). IF samples were mounted with VectaShield Hardset with DAPI mounting media (Vector, Burlingame, CA, USA).

### Human KRAS2 amplification assay

We performed real-time quantitative PCR and fluorescent melting curve analyses using genomic DNAs from 16 basal-like tumors, a normal breast tissue sample, 2 leukocyte DNA, and 3 luminal tumors. DNA was extracted using the DNAeasy kit (Qiagen) and amplification was performed on the LightCycler using the following temperature parameters: 95°C, 8 minutes; 50 cycles of 57°C, 6 s; 72°C, 6 s; 95°C, 2 s; followed by cooling to 60°C and a 0.1°C/s ramp to 97°C. Each PCR reaction contained 7.5 ng template DNA in a 10 μl reaction using the LightCycler Faststart DNA Master SYBR Green I kit (Roche Applied Science, Indianapolis, IN, USA). Relative DNA copy number for each gene was determined by importing an external efficiency curve and using a 'normal' breast sample for a within-run calibrator. For each sample, the copy number for KRAS2 was divided by the average copy number of ACTB and G1P3. Amplification in any tumor was called if the relative fold change was greater than three standard deviations above the average of five control samples (two normal leukocyte samples and three luminal tumors).

## Additional data files

The following additional data are available with the online version of this paper. Additional data file 1 is a table listing mouse tumor and normal sample associated data, including source, transgene and promoter information. Additional data file 2 is a complete unsupervised cluster diagram of all mouse tumors. Samples are colored according to mouse model from which they were derived, and the genes were selected using a variation filter of three-fold or more on three or more samples. Additional data file 3 ia a complete mouse models cluster diagram using the 866 gene murine intrinsic gene list. Additional data file 4 provides CC analyses applied to the mouse models. **(a) **CC matrices generated using the 866 gene mouse intrinsic list, by cluster numbers K = 2 through K = 15. **(b) **Empirical cumulative distribution (CDF) plot corresponding to the consensus matrices in the range K = 2 to 15. **(c) **CC directly compared to the hierarchical clustering-based results. The dendrogram from Figure 1 (using the intrinsic gene set) is shown and immediately below is a colored matrix showing sample assignments based upon the various number of K clusters from the CC. By comparison, the analysis performed on the mouse dataset using all genes (bottom matrix) is presented. Additional data file 5 is a complete unsupervised cluster diagram of the combined gene expression patterns of 232 human breast tumor samples and 122 mouse mammary tumor samples. This unsupervised cluster analysis is based upon the orthologous gene overlap between the human and mouse microarrays, and then we selected for the subset of genes that varied three-fold or more on three or more arrays. Additional data file 6 shows a cluster analysis of the 232 human samples using the human intrinsic/UNC gene set from Hu *et al*. [21]. This analysis was used to determine a human samples subtype (basal-like, luminal, HER2+/ER-, and so on), which was then used in the various SAM and GSEA analyses. Samples are colored according to their subtype: red = basal-like, blue = luminal, pink = HER2+/ER-, yellow = claudin-low and green = normal breast-like. Additional data file 7 shows a histological characterization of six different human 'claudin-low' tumors using hematoxylin and eosin sections. Additional data file 8 shows GSEA of murine pathway models versus five human subtypes. Additional data file 9 shows GSEA of ten murine classes versus clinical ER status and HER2 status in ER negative patients. Additional data file 10 shows GSEA of murine pathway models versus clinical ER status and HER2 status in ER negative patients.

## Supplementary Material

Additional data file 1 Mouse tumor and normal sample associated data including source, transgene and promoter information. Click here for file

Additional data file 2 Samples are colored according to mouse model from which they were derived, and the genes were selected using a variation filter of three-fold or more on three or more samples. Click here for file

Additional data file 3 Complete mouse models cluster diagram using the 866 gene murine intrinsic gene list. Click here for file

Additional data file 4 **(a) **CC matrices generated using the 866 gene mouse intrinsic list, by cluster numbers K = 2 through K = 15. **(b) **Empirical cumulative distribution (CDF) plot corresponding to the consensus matrices in the range K = 2 to 15. **(c) **CC directly compared to the hierarchical clustering-based results. The dendrogram from Figure 1 (using the intrinsic gene set) is shown and immediately below is a colored matrix showing sample assignments based upon the various number of K clusters from the CC. By comparison, the analysis performed on the mouse dataset using all genes (bottom matrix) is presented. Click here for file

Additional data file 5 This unsupervised cluster analysis is based upon the orthologous gene overlap between the human and mouse microarrays, and then we selected for the subset of genes that varied three-fold or more on three or more arrays. Click here for file

Additional data file 6 This analysis was used to determine a human samples subtype (basal-like, luminal, HER2+/ER-, and so on), which was then used the various SAM and GSEA analyses. Samples are colored according to their subtype: red = basal-like, blue = luminal, pink = HER2+/ER-, yellow = claudin-low and green = normal breast-like. Click here for file

Additional data file 7 Histological characterization of six different human 'claudin-low' tumors using hematoxylin and eosin sections. Click here for file

Additional data file 8 GSEA of murine pathway models versus five human subtypes. Click here for file

Additional data file 9 GSEA of ten murine classes versus clinical ER status and HER2 status in ER negative patients. Click here for file

Additional data file 10 GSEA of murine pathway models versus clinical ER status and HER2 status in ER negative patients. Click here for file
