# Supplementary figures and images for: Identification of conserved gene expression features between murine mammary carcinoma models and human breast tumors
Source: Genome Biol. 2007 May 10;8(5):R76. doi: 10.1186/gb-2007-8-5-r76 (PMC1929138; doi:10.1186/gb-2007-8-5-r76)

Additional Data File 2

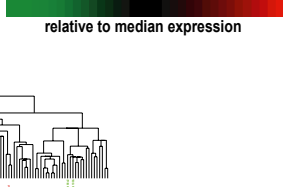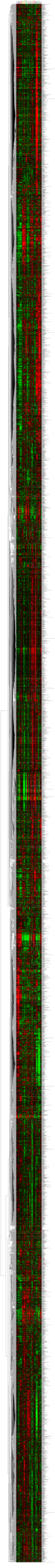

Supplement: Additional data file 2 — Samples are colored according to mouse model from which they were derived, and the genes were selected using a variation filter of three-fold or more on three or more samples. [file gb-2007-8-5-r76-S2.pdf]

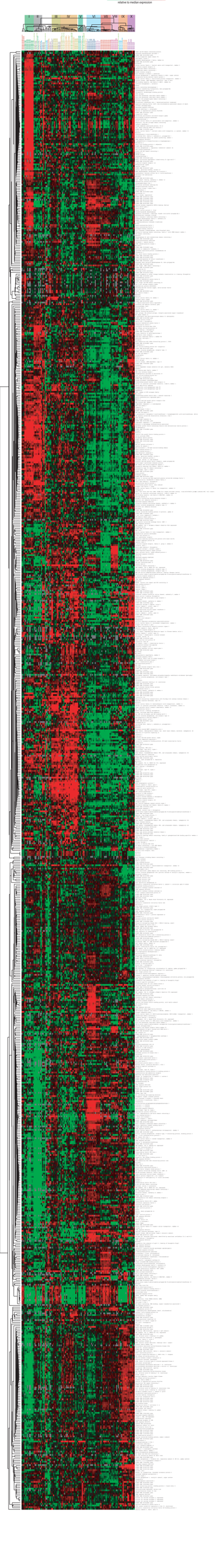

Supplement: Additional data file 3 — Complete mouse models cluster diagram using the 866 gene murine intrinsic gene list. [file gb-2007-8-5-r76-S3.pdf]

Additional Data File 5

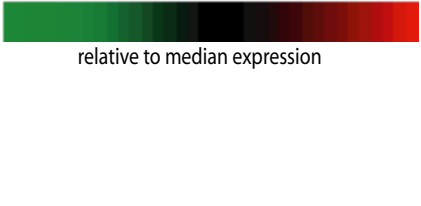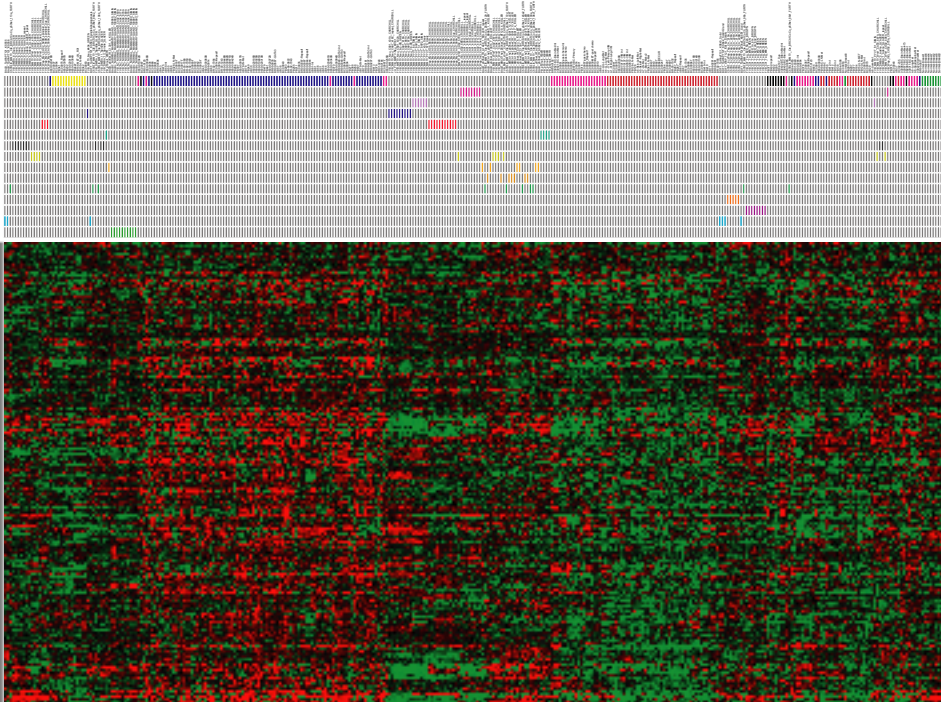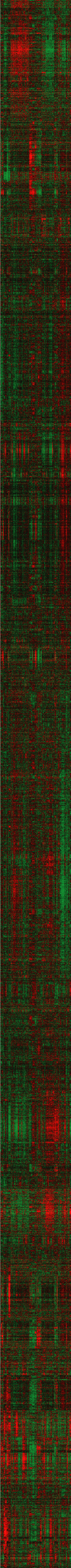

Supplement: Additional data file 5 — This unsupervised cluster analysis is based upon the orthologous gene overlap between the human and mouse microarrays, and then we selected for the subset of genes that varied three-fold or more on three or more arrays. [file gb-2007-8-5-r76-S5.pdf]

# Additional Data File 6

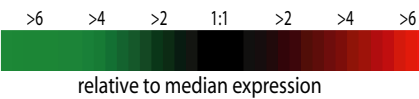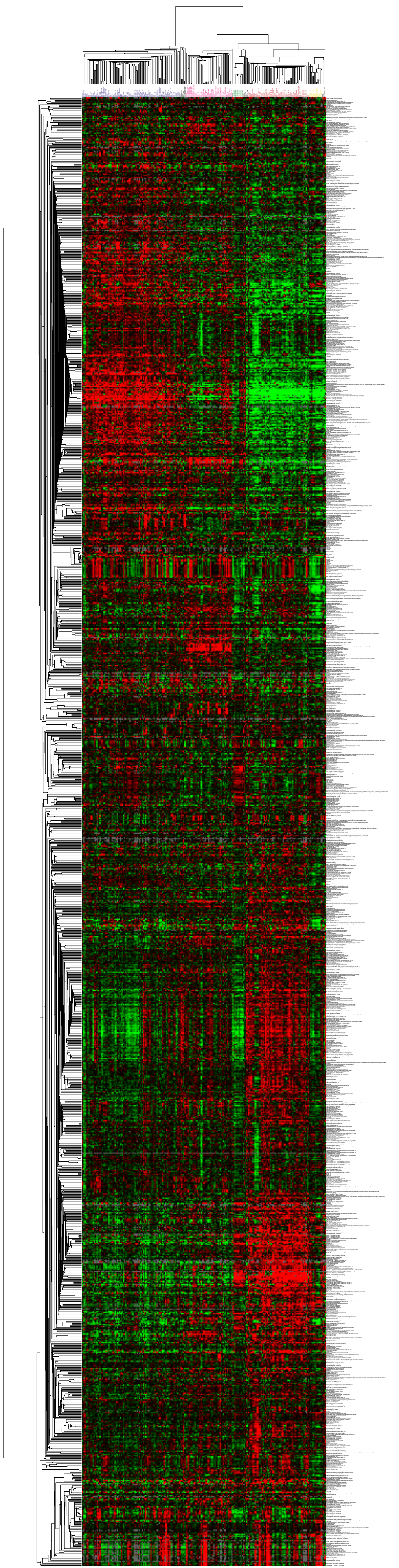

Supplement: Additional data file 6 — This analysis was used to determine a human samples subtype (basal-like, luminal, HER2+/ER-, and so on), which was then used the various SAM and GSEA analyses. Samples are colored according to their subtype: red = basal-like, blue = luminal, pink = HER2+/ER-, yellow = claudin-low and green = normal breast-like. [file gb-2007-8-5-r76-S6.pdf]

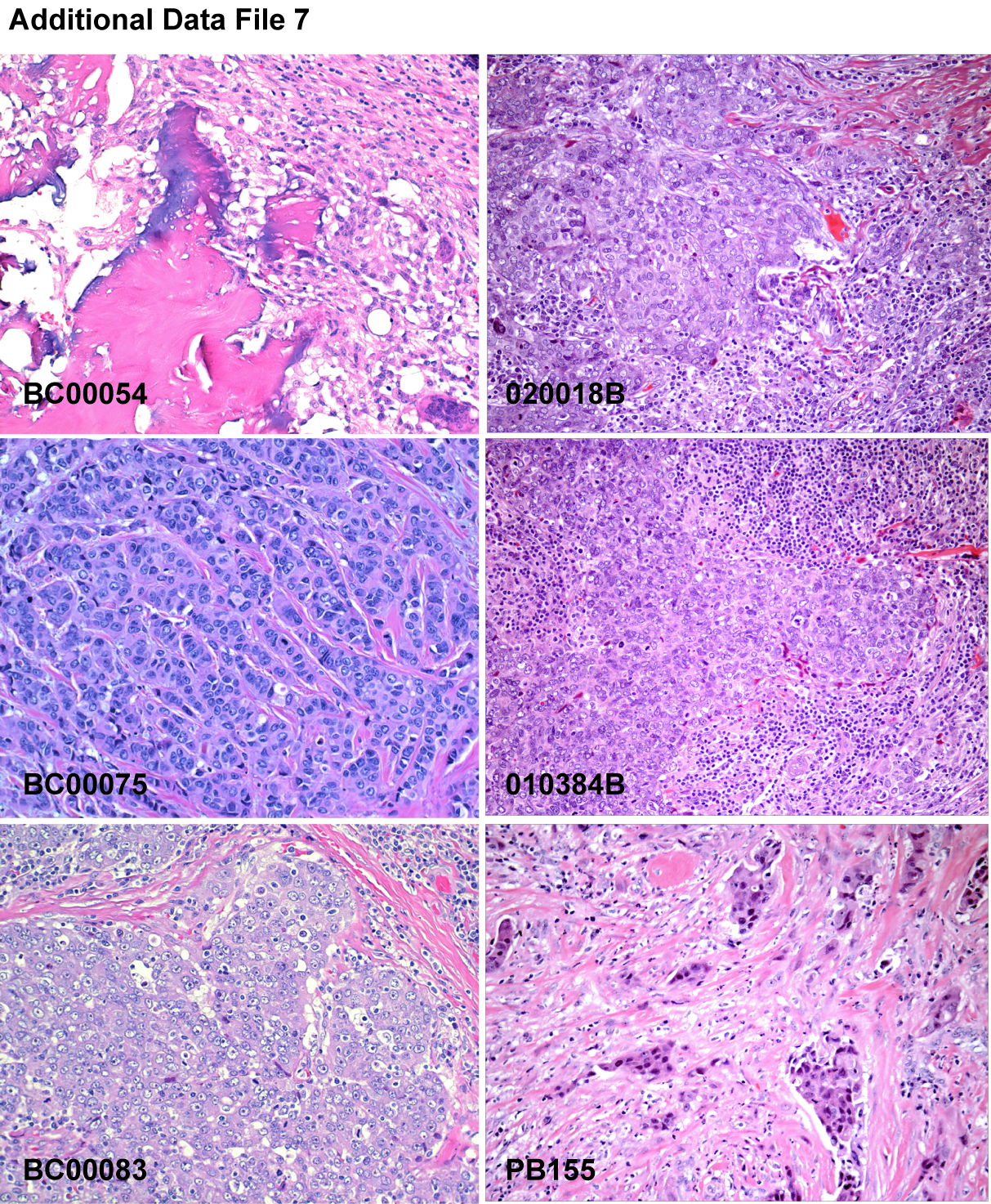

Supplement: Additional data file 7 — Histological characterization of six different human 'claudin-low' tumors using hematoxylin and eosin sections. [file gb-2007-8-5-r76-S7.tiff]
