# Supplementary material for: Identification of conserved gene expression features between murine mammary carcinoma models and human breast tumors
Source: Genome Biol. 2007 May 10;8(5):R76. doi: 10.1186/gb-2007-8-5-r76 (PMC1929138; doi:10.1186/gb-2007-8-5-r76)
Supplement: Additional data file 4 — (a) CC matrices generated using the 866 gene mouse intrinsic list, by cluster numbers K = 2 through K = 15. (b) Empirical cumulative distribution (CDF) plot corresponding to the consensus matrices in the range K = 2 to 15. (c) CC directly compared to the hierarchical clustering-based results. The dendrogram from Figure 1 (using the intrinsic gene set) is shown and immediately below is a colored matrix showing sample assignments based upon the various number of K clusters from the CC. By comparison, the analysis performed on the mouse dataset using all genes (bottom matrix) is presented. [file gb-2007-8-5-r76-S4.pdf]

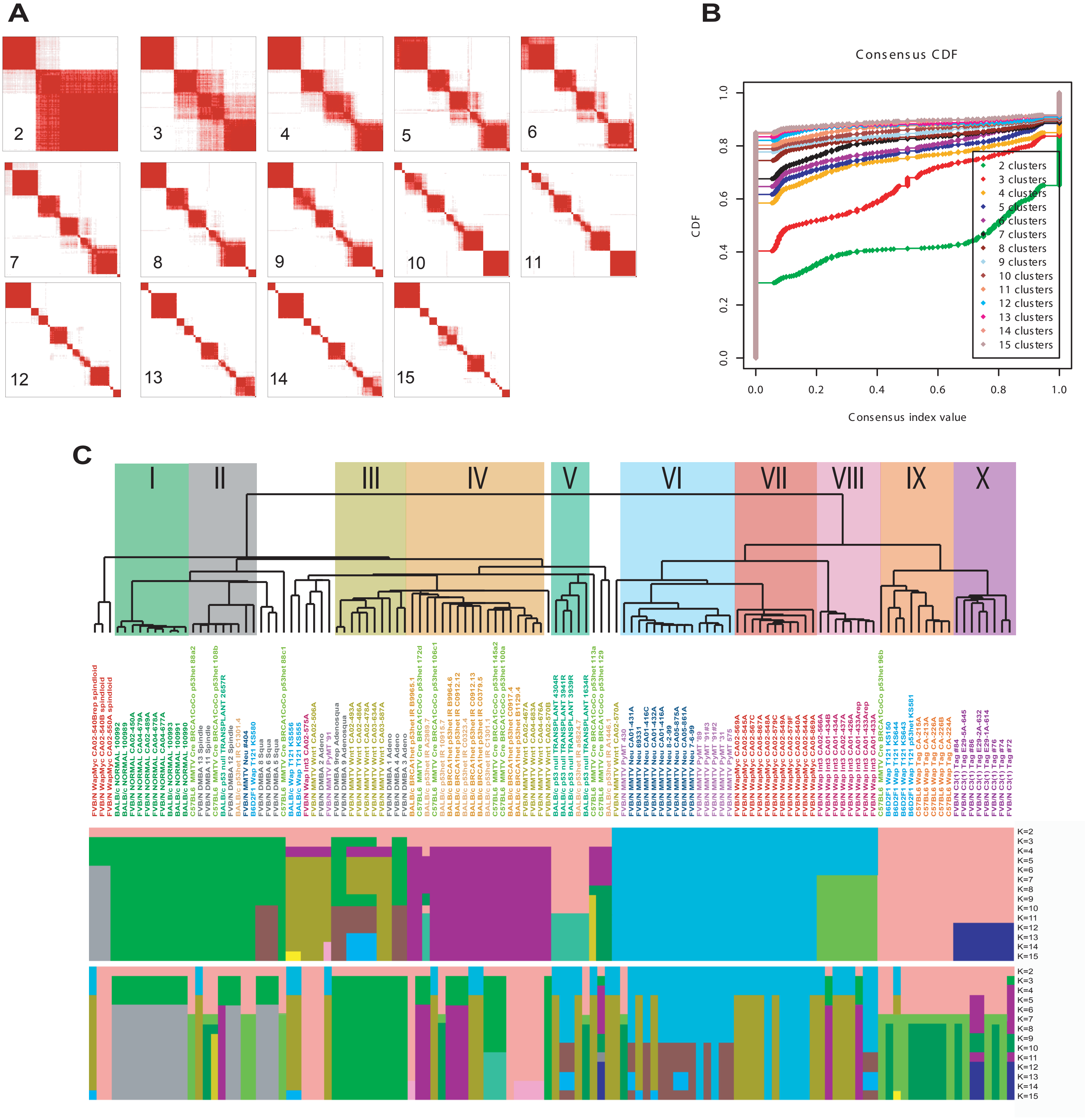
A
2
3
4
5
6
7
8
9
10
11
12
13
14
15
B
Consensus CDF
CDF
Consensus index value
0.0
0.2
0.4
0.6
0.8
1.0
2 clusters
3 clusters
4 clusters
5 clusters
6 clusters
7 clusters
8 clusters
9 clusters
10 clusters
11 clusters
12 clusters
13 clusters
14 clusters
15 clusters
C
I
II
III
IV
V
VI
VII
VIII
IX
X
K=2
K=3
K=4
K=5
K=6
K=7
K=8
K=9
K=10
K=11
K=12
K=13
K=14
K=15
