# Supplementary material for: Identification of conserved gene expression features between murine mammary carcinoma models and human breast tumors
Source: Genome Biol. 2007 May 10;8(5):R76. doi: 10.1186/gb-2007-8-5-r76 (PMC1929138; doi:10.1186/gb-2007-8-5-r76)
Supplement: Additional data file 8 — GSEA of murine pathway models versus five human subtypes. [file gb-2007-8-5-r76-S8.doc]

**Additional Data File 8.** Gene Set Enrichment Analysis (GSEA) of murine pathway models versus 5 human subtypes. Statistically significant findings are highlighted in bold.

| **Is Class** | | | | | | | | | | | |
| --- | --- | --- | --- | --- | --- | --- | --- | --- | --- | --- | --- |
|  |  | **BASAL-like** | | **LUMINAL** | | **HER2+/ER-** | | **NORMAL** | | **claudin-LOW** | |
| **Mouse Model** | # genes | NOM p-val | FWER p-val | NOM p-val | FWER p-val | NOM p-val | FWER p-val | NOM p-val | FWER p-val | NOM p-val | FWER p-val |
| T-antigen models | 406 | 0 | **0.002** | - | - | - | - | - | - | - | - |
| BRCA1 models | 427 | 0.004 | 0.0881 | - | - | - | - | - | - | - | - |
| Wnt1 model | 34 | 0.1159 | 0.5215 | - | - | 0.1741 | 0.6356 | 0.0629 | 0.2553 | - | - |
| Myc.model | 517 | 0.1438 | 0.6086 | - | - | 0.4553 | 0.948 | - | - | - | - |
| BRCA1 p53 IR model | 65 | 0.1957 | 0.6867 | - | - | - | - | 0.3354 | 0.8739 | - | - |
| Int3 model | 137 | 0.283 | 0.8428 | - | - | 0.1706 | 0.7417 | 0.0278 | 0.3904 | - | - |
| Neu.model | 460 | - | - | 0.0061 | 0.154 | 0.1594 | 0.6847 | 0.9014 | 0.997 | - | - |
|  |  |  |  |  |  |  |  |  |  |  |  |
|  |  |  |  |  |  |  |  |  |  |  |  |
| **Is Not Class** | | | | | | | | | | | |
|  |  | **BASAL-like** | | **LUMINAL** | | **HER2+/ER-** | | **NORMAL** | | **claudin-LOW** | |
| **Mouse Model** | # genes | NOM p-val | FWER p-val | NOM p-val | FWER p-val | NOM p-val | FWER p-val | NOM p-val | FWER p-val | NOM p-val | FWER p-val |
| T-antigen models | 406 | - | - | 0.043 | 0.087 | 0.899 | 0.999 | 0.06831 | 0.207 | 0.5962 | 0.996 |
| BRCA1 models | 427 | - | - | 0.1037 | 0.52 | 0.5203 | 0.968 | 0.14 | 0.605 | 0.7519 | 0.997 |
| Wnt1 model | 34 | - | - | 0.01734 | 0.113 | - | - | - | - | 0.2417 | 0.787 |
| Myc.model | 517 | - | - | 0.126 | 0.557 | - | - | 0.7692 | 0.998 | 0.278 | 0.85 |
| BRCA1 p53 IR model | 65 | - | - | 0.5198 | 0.966 | 0.6536 | 0.991 | - | - | 0.1063 | 0.499 |
| Int3 model | 137 | - | - | 0.1996 | 0.799 | - | - | - | - | 0.2079 | 0.83 |
| Neu.model | 460 | 0 | **0.027** | - | - | - | - | - | - | 0.4188 | 0.939 |
