# Supplementary material for: Identification of conserved gene expression features between murine mammary carcinoma models and human breast tumors
Source: Genome Biol. 2007 May 10;8(5):R76. doi: 10.1186/gb-2007-8-5-r76 (PMC1929138; doi:10.1186/gb-2007-8-5-r76)
Supplement: Additional data file 9 — GSEA of ten murine classes versus clinical ER status and HER2 status in ER negative patients. [file gb-2007-8-5-r76-S9.doc]

**Additional Data File 9.** Gene Set Enrichment Analysis (GSEA) of 10 murine classes versus clinical ER status and HER2 status in ER negative patients. Statistically significant findings are highlighted in bold.

| **Is Class** | | | | | | | |
| --- | --- | --- | --- | --- | --- | --- | --- |
|  |  | **ER +** | | **ER- / HER2-** | | **ER- / HER2+** | |
| **Mouse Class** | # genes | NOM p-val | FWER p-val | NOM p-val | FWER p-val | NOM p-val | FWER p-val |
| I | 1882 | 0.2267 | 0.795 | - | - | 0.9243 | 0.997 |
| II | 912 | - | - | - | - | 0.5675 | 0.988 |
| III | 143 | 0.7919 | 0.999 | - | - | 0.2723 | 0.837 |
| IV | 1019 | - | **-** | 0.0062 | 0.323 | - | - |
| V | 34 | - | - | 0.668 | 0.994 | 0.4929 | 0.964 |
| VI | 820 | 0.0098 | 0.174 | - | - | 0.6111 | 0.976 |
| VII | 851 | - | - | 0.1417 | 0.618 | 0.5666 | 0.975 |
| VIII | 236 | 0.3379 | 0.922 | - | - | 0.1244 | 0.73 |
| IX | 462 | - | **-** | 0 | **0.009** | 0.8112 | 0.998 |
| X | 338 | - | **-** | 0 | **0.003** | - | - |
|  |  |  |  |  |  |  |  |
|  |  |  |  |  |  |  |  |
| **Is Not Class** | | | | | | | |
|  |  | **ER +** | | **ER- / HER2-** | | **ER- / HER2+** | |
| **Mouse Class** | # genes | NOM p-val | FWER p-val | NOM p-val | FWER p-val | NOM p-val | FWER p-val |
| I | 1882 | - | - | 0.0829 | 0.522 | - | - |
| II | 912 | 0.7182 | 0.999 | 0.7589 | 1 | - | - |
| III | 143 | - | - | 0.5337 | 0.978 | - | - |
| IV | 1019 | 0.012 | 0.469 | - | - | 0.3107 | 0.909 |
| V | 34 | 0.5571 | 0.985 | - | - | - | - |
| VI | 820 | - | **-** | 0.0041 | 0.061 | - | - |
| VII | 851 | 0.1726 | 0.675 | - | - | - | - |
| VIII | 236 | - | - | 0.2432 | 0.873 | - | - |
| IX | 462 | 0.00641 | **0.024** | - | - | - | - |
| X | 338 | 0.00641 | **0.043** | - | - | 0.6922 | 0.999 |
