# Supplementary material for: Identification of conserved gene expression features between murine mammary carcinoma models and human breast tumors
Source: Genome Biol. 2007 May 10;8(5):R76. doi: 10.1186/gb-2007-8-5-r76 (PMC1929138; doi:10.1186/gb-2007-8-5-r76)
Supplement: Additional data file 10 — GSEA of murine pathway models versus clinical ER status and HER2 status in ER negative patients. [file gb-2007-8-5-r76-S10.doc]

**Additional Data File 10.** Gene Set Enrichment Analysis (GSEA) of murine pathway models versus clinical ER status and HER2 status in ER negative patients. Statistically significant findings are highlighted in bold.

| **Is Class** | | | | | | | |  |
| --- | --- | --- | --- | --- | --- | --- | --- | --- |
|  |  | **ER +** | | **ER- / HER2-** | | **ER- / HER2+** | | |
| **Mouse Model** | # genes | NOM p-val | FWER p-val | NOM p-val | FWER p-val | NOM p-val | FWER p-val | |
| T-antigen models | 406 | - | - | 0 | **0.003** | - | - | |
| BRCA1 models | 427 | - | - | 0.0061 | 0.116 | 0.7755 | 0.993 | |
| Wnt1 model | 34 | - | - | 0.4073 | 0.938 | 0.2478 | 0.775 | |
| Myc.model | 517 | - | **-** | 0.1253 | 0.573 | 0.5506 | 0.975 | |
| BRCA1 p53 IR model | 65 | - | - | 0.6159 | 0.993 | 0.5648 | 0.971 | |
| Int3 model | 137 | | 0.1988 | | --- | | 0.818 | - | - | - | - | |
| Neu.model | 460 | 0.0078 | 0.185 | - | - | 0.5479 | 0.965 | |
|  |  |  |  |  |  |  |  | |
|  |  |  |  |  |  |  |  | |
| **Is Not Class** | | | | | | | |  |
|  |  | **ER +** | | **ER- / HER2-** | | **ER- / HER2+** | | |
| **Mouse Model** | # genes | NOM p-val | FWER p-val | NOM p-val | FWER p-val | NOM p-val | FWER p-val | |
| T-antigen models | 406 | 0.0106 | **0.022** | - | - | 0.8453 | 1 | |
| BRCA1 models | 427 | 0.0098 | 0.17 | - | - | - | - | |
| Wnt1 model | 34 | 0.3252 | 0.858 | - | - | - | - | |
| Myc.model | 517 | 0.1206 | 0.604 | - | - | - | - | |
| BRCA1 p53 IR model | 65 | 0.6063 | 0.985 | - | - | - | - | |
| Int3 model | 137 | - | **-** | 0.3202 | 0.899 | 0.5552 | 0.978 | |
| Neu.model | 460 | - | - | 0.002 | 0.079 | - | - | |
